# Dynamic *Arabidopsis* P5CS filament facilitates substrate channelling

**DOI:** 10.1038/s41477-024-01697-w

**Published:** 2024-05-13

**Authors:** Chen-Jun Guo, Tianyi Zhang, Qingqing Leng, Xian Zhou, Jiale Zhong, Ji-Long Liu

**Affiliations:** 1 School of Life Science and Technology, ShanghaiTech University, Shanghai, China; 2 Department of Physiology, Anatomy and Genetics, University of Oxford, Oxford, UK; 3 Shanghai Clinical Research and Trial Center, Shanghai, China

**Keywords:** Plant molecular biology, Plant breeding

## Abstract

In plants, the rapid accumulation of proline is a common response to combat abiotic stress^1–7^. Delta-1-pyrroline-5-carboxylate synthase (P5CS) is a rate-limiting enzyme in proline synthesis, catalysing the initial two-step conversion from glutamate to proline^8^. Here we determine the first structure of plant P5CS. Our results show that *Arabidopsis thaliana* P5CS1 (AtP5CS1) and P5CS2 (AtP5CS2) can form enzymatic filaments in a substrate-sensitive manner. The destruction of AtP5CS filaments by mutagenesis leads to a significant reduction in enzymatic activity. Furthermore, separate activity tests on two domains reveal that filament-based substrate channelling is essential for maintaining the high catalytic efficiency of AtP5CS. Our study demonstrates the unique mechanism for the efficient catalysis of AtP5CS, shedding light on the intricate mechanisms underlying plant proline metabolism and stress response.

## Abstract (web summary)

Abiotic stresses challenge plant growth. In response, plants often rapidly accumulate proline. This study reveals the filament structures of plant P5CS, the key enzyme in proline synthesis, elucidating a unique mechanism for its efficient catalysis.

## Main

**Extended Data Fig. 1:**
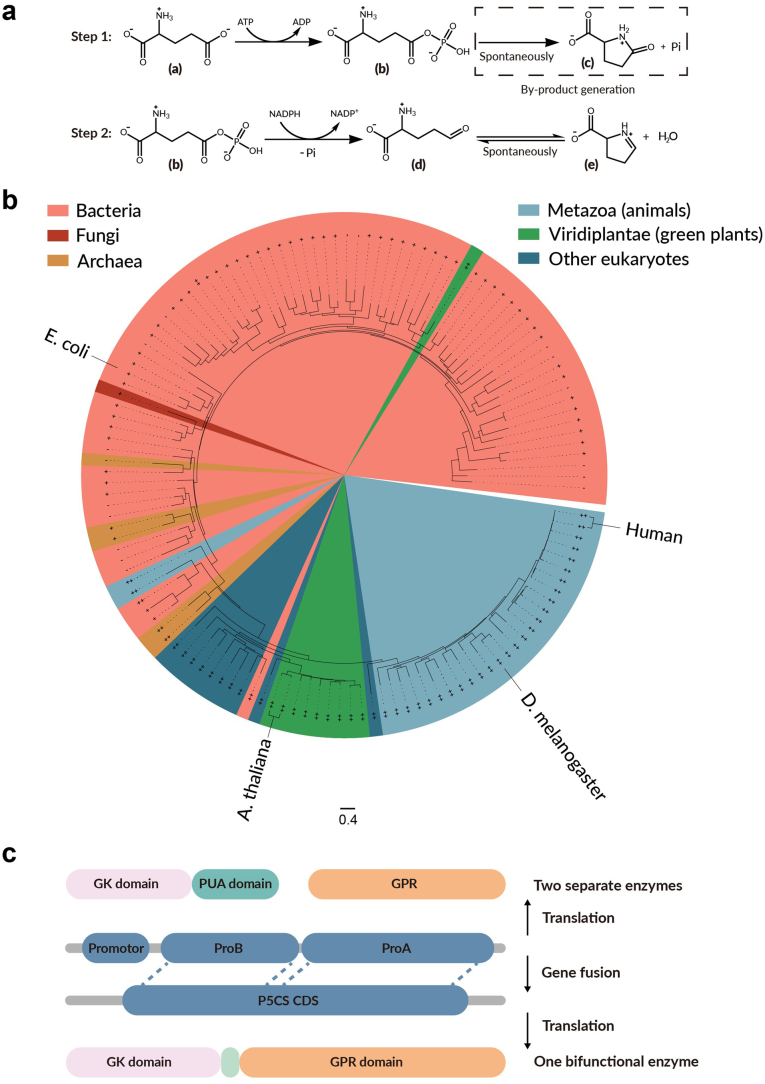
Function and evolution of GK and GPR. (**a**) Two-step reaction catalyzed by GK and GPR. A possible side reaction is shown in the dashed box. a: L-glutamate. b: G5P. c: GSA. d: L-Pyroglutamic acid. e: Delta-1-pyrroline-5-carboxylate. (**b**) Phylogenetic tree of GK and P5CS proteins. Domain organization of these GK and P5CS is represented with marker at the extension of each clade, where ‘−’ represent GK without PUA domain, ‘+’ represent GK with PUA domain, and ‘++’ represent P5CS. (**c**) Scheme of the fusion of GK and GPR during evolution.

Delta-1-pyrroline-5-carboxylate synthase (P5CS) is a key enzyme in proline biosynthesis pathway. This enzyme catalyses a two-step reaction that converts glutamate to l-glutamate-5-phosphate (G5P) and then to l-glutamate-5-semialdehyde. l-Glutamate-5-semialdehyde will spontaneously cyclize and reach equilibrium with delta-1-pyrroline-5-carboxylate (P5C) (Extended Data Fig. 1a). The final product P5C is an essential precursor for proline biosynthesis^8^. As a bifunctional enzyme, P5CS has two structural domains: the glutamate kinase (GK) domain and the glutamyl phosphate reductase (GPR) domain. In prokaryotes, GK and GPR are separate enzymes, and in most eukaryotes they are fused into a bifunctional P5CS^9^ (Extended Data Fig. 1b,c and Supplementary Fig. 1). We have found that *Drosophila melanogaster* P5CS (DmP5CS) can form cytoophidium, and purified DmP5CS can also form metabolic filaments in vitro^10^. It is uncertain whether filamentation of P5CS is a conserved phenomenon or a special case of a few species. Except for DmP5CS, the full-length structure of P5CS has not been reported.

**Fig. 1:**
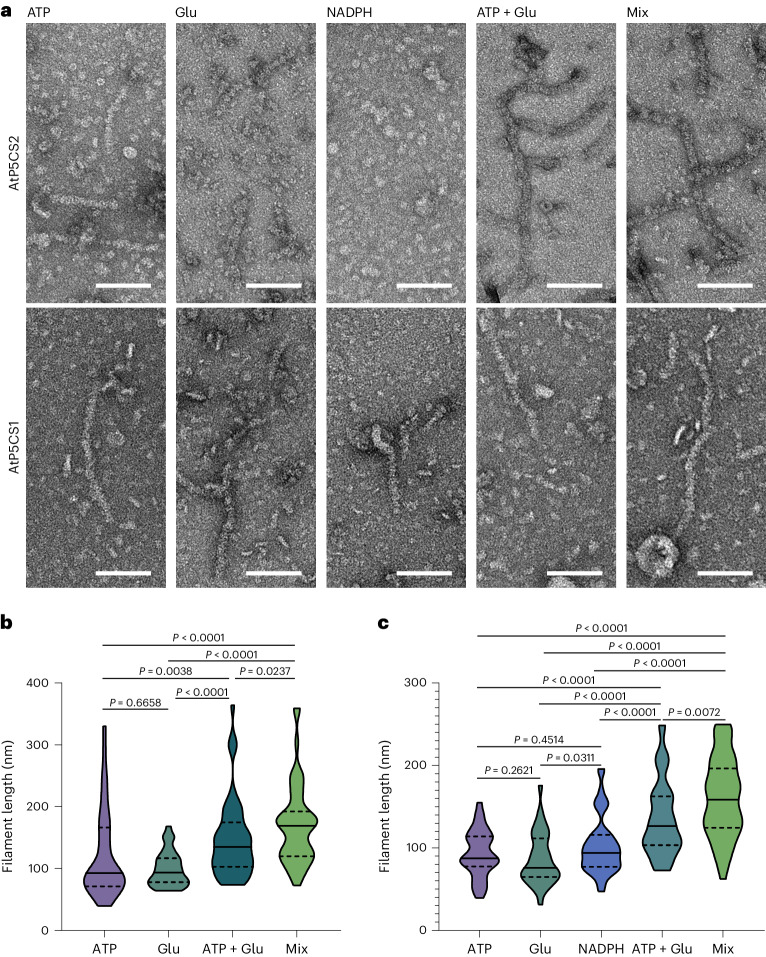
Both AtP5CS1 and AtP5CS2 form active filaments in vitro and respond to the ligands. **a**, Negative-staining EM micrographs of AtP5CS2 and AtP5CS1 incubated with different substrate combinations. Scale bars, 100 nm. **b**, Statistical analysis of AtP5CS2 filament length under different substrate conditions. Truncated violin plots show the medians (solid lines) and quartiles (dashed lines). Two-sided Mann–Whitney test was used. **c**, Statistical analysis of AtP5CS1 filament length under different substrate conditions. Truncated violin plots show the medians (solid lines) and quartiles (dashed lines). Two-sided Mann–Whitney test was used.

**Extended Data Fig. 3:**
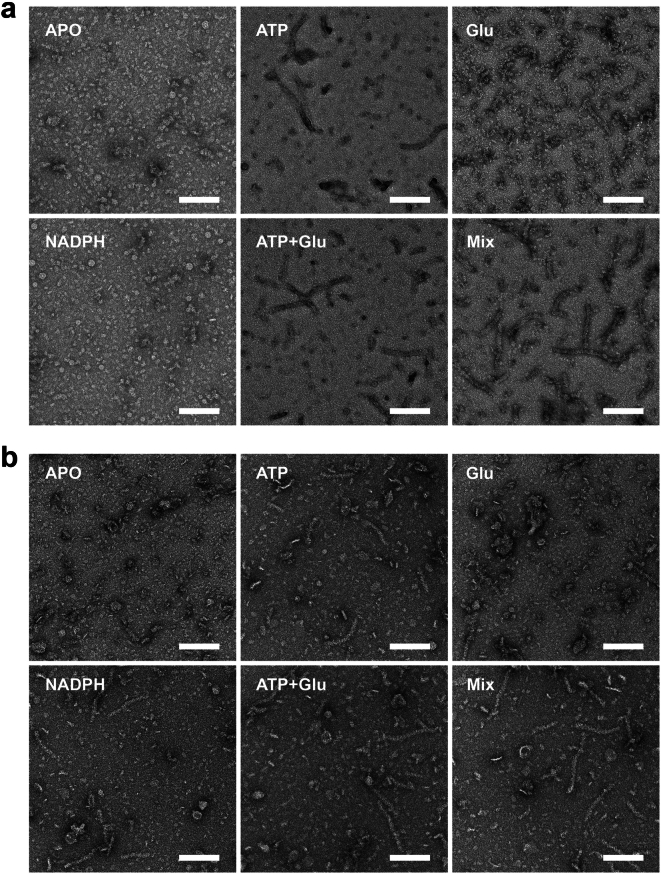
Representative negative staining images of AtP5CS. Negative staining images of (**a**) AtP5CS2 and (**b**) AtP5CS1 incubated with different substrates. Scale bar: 150 nm. The negative staining experiments have been repeated for 3 times with similar results and each time more than ten different areas were checked.

In this Letter, we choose AtP5CS as the model to investigate the structure and function of plant P5CS. The two P5CSs in *Arabidopsis thaliana*, AtP5CS1 and AtP5CS2, share 89% identity in protein sequence. AtP5CS2 is easily expressed in *Escherichia coli* with Small Ubiquitin-like Modifier (SUMO)-tag. During purification, AtP5CS2 showed a wide range of molecular weights in size exclusion chromatography (Supplementary Fig. 2). The oligomerization state of AtP5CS2 was checked with negative-staining electron microscopy (EM). At APO state (ligand-free state) (with 20 mM Tris–HCl, 150 mM NaCl, 10 mM MgCl_2_, pH = 8.0), no filament was observed (Extended Data Fig. 3a). At P5CS2, filaments will appear once there is adenosine triphosphate (ATP) or glutamate in the buffer (Fig. 1a). When both ATP and glutamate are present, longer filaments were observed (Fig. 1b). The average length of AtP5CS2 filaments increased significantly when all substrates are added (mixed state) compared with all other conditions (Fig. 1b).

After numerous attempts, we were unable to obtain a stable, active AtP5CS1 protein through the SUMO-tag expression system. Consequently, we explored direct expression using the N-terminal 6His system and successfully acquired active AtP5CS1 protein (Supplementary Fig. 3). However, application of size exclusion chromatography led to the inactivation of AtP5CS1, and we only used one-step affinity purification for AtP5CS1. To avoid the affection of glycerol on the following cryo-EM study, we opted for a low-salt buffer without glycerol for protein elution.

Upon examination through negative-staining EM, we discovered the filamentation of AtP5CS1 showed a pattern similar to that of AtP5CS2 (Fig. 1a,c and Extended Data Fig. 3). Interestingly, under conditions with only reduced nicotinamide adenine dinucleotide phosphate (NADPH) present, the AtP5CS1 sample showed a capacity for filament formation, while AtP5CS2 maintained its oligomeric state. With these negative staining and statistical data, we conclude both AtP5CS1 and AtP5CS2 could form filaments, and this filamentation is regulated by their substrates. Compared with DmP5CS, the regulation of AtP5CS filament is different. For DmP5CS, the contribution of glutamate is greater than that of ATP^11^. For AtP5CS1 and AtP5CS2, both glutamate and ATP promote filament formation at similar degrees (Fig. 1b,c). Both DmP5CS and AtP5CS form the longest filaments at mixed state.

**Fig. 2:**
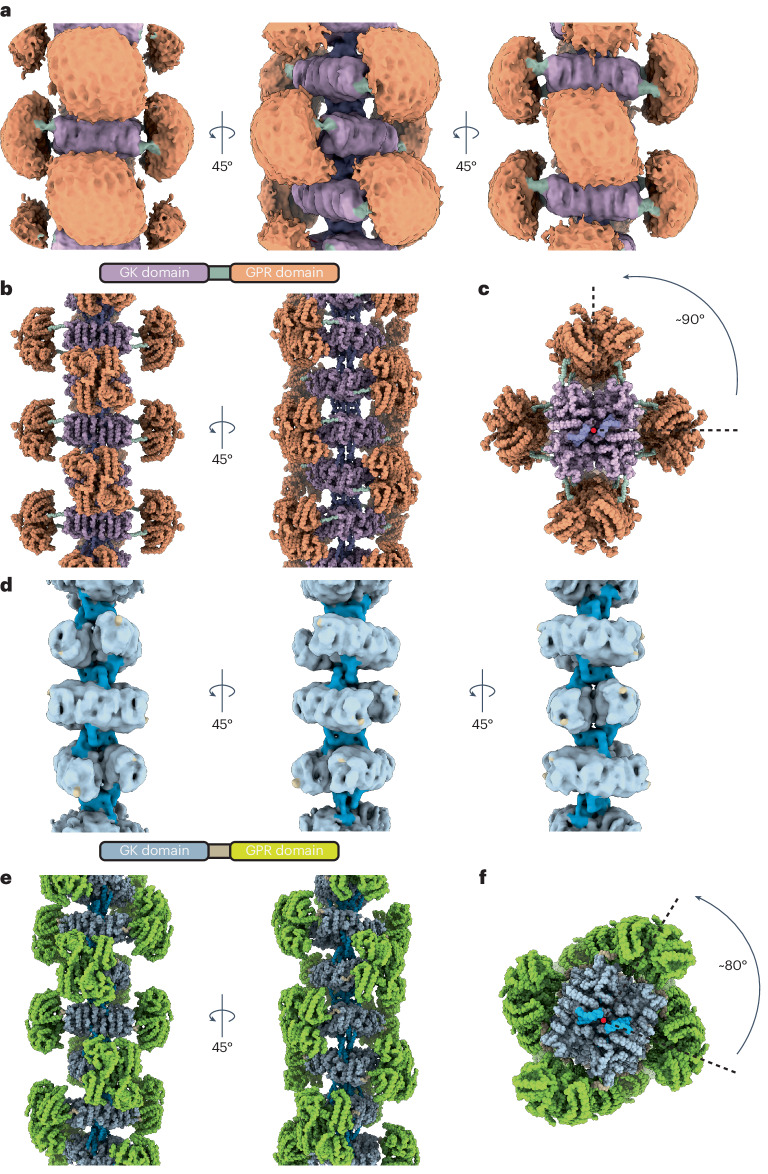
Overall structure of AtP5CS filaments. **a**, Density map of AtP5CS2 filament. GK domain, hook, GPR domain and linker of AtP5CS2 filament are coloured differently. **b**, Proposed model for the AtP5CS2 filament. This model was generated by using the predicted full-length model of AtP5CS2 from AlphaFold2 and fitting it into our density map. **c**, The top view of the AtP5CS2 filament. **d**, Density map of AtP5CS1 filament. GK domain, hook, GPR domain and linker of AtP5CS1 filament are coloured differently. **e**, Proposed model for the AtP5CS1 filament. This model was generated by using the predicted full-length model of AtP5CS1 from AlphaFold2 and fitting it into our density map. **f**, The top view of the AtP5CS1 filament.

**Extended Data Fig. 4:**
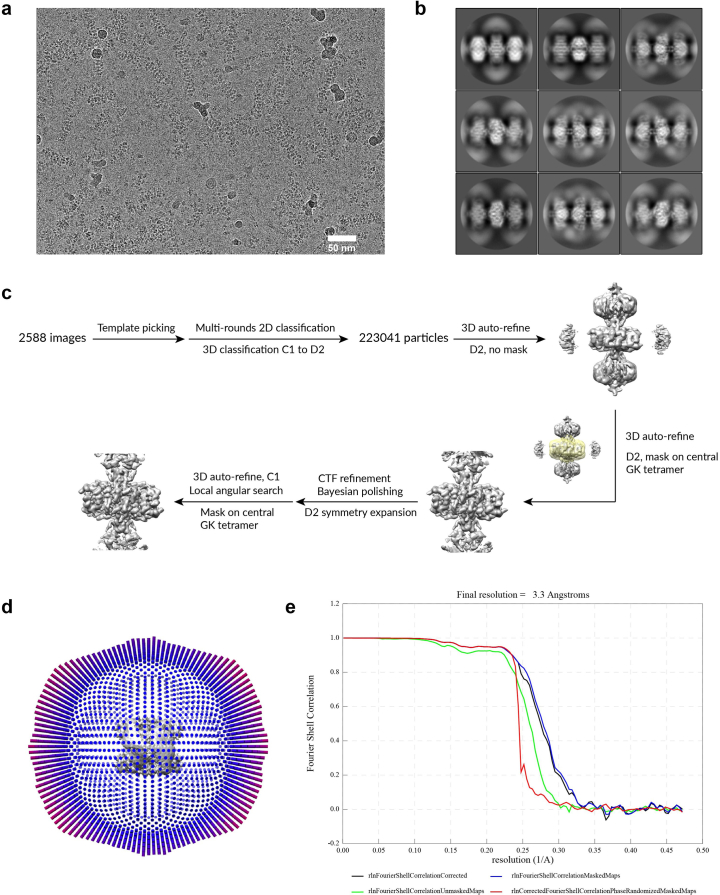
Data processing of AtP5CS2. (**a**) Representative micrograph (from 2588 micrographs) after motion correction. (**b**) Representative 2D classification results. (**c**) Workflow for reconstruction AtP5CS2 filament and focus refinement of GK tetramer. (**d**) Angular distribution (top view) of final GK tetramer reconstruction. Particles had been D2 expanded. (**e**) FSC curves for focus refined GK tetramer.

We first determined the cryo-EM structure of mixed-state AtP5CS2 (Extended Data Fig. 4 and Supplementary Table 1). Using single-particle analysis and focus refinement strategy, we obtained a map containing about five helical units of AtP5CS2 (Fig. 2a). Each helical unit is a tetramer, in which the GK domain is located at the core as one tetramer and the GPR domain is located on both sides as two dimers. Helical units are arranged in a perpendicular way, and an obvious interface consists of hooks located on the helical axis. Interestingly, in our map, there is no apparent interaction between adjacent GPRs, which is thought to be important for the activity of DmP5CS.

The only connection between the GK domain and the GPR domain is a flexible linker, and large motion is observed between the GK tetramer and the GPR dimer. In the overall reconstruction, only a blob can be seen at the GPR domain. After focus refinement, the central GK tetramer could reach an average 3.3 Å resolution (Extended Data Fig. 4), but no high-resolution reconstruction for the GPR domain was made. By fitting the full-length model generated by AlphaFold2 into our map and with manual adjustments, we built the model for AtP5CS2 filament (Fig. 2b). The hook between adjacent helical units can be seen clearly in the side view of this model, and the whole filament appears as a cross in the top view due to the orthogonal assembly of AtP5CS2 (Fig. 2c).

**Extended Data Fig. 5:**
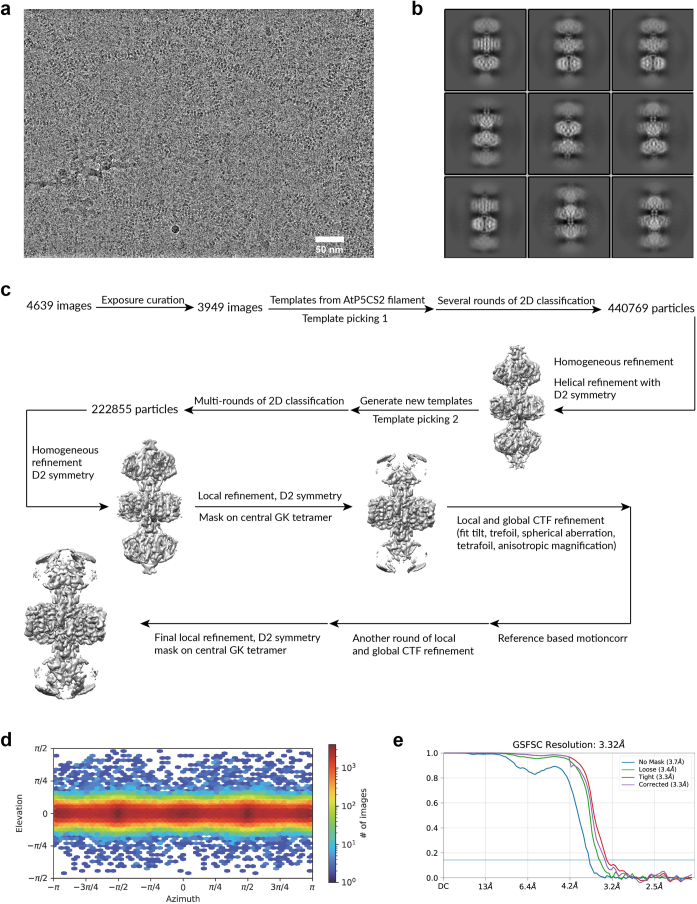
Data processing of AtP5CS1. (**a**) Representative micrograph (from 4639 micrographs) after motion correction. (**b**) Representative 2D classification results. (**c**) Workflow for reconstruction AtP5CS1 filament and focus refinement of GK tetramer. (**d**) Angular distribution of focus refined GK tetramer reconstruction. (**e**) FSC curves for focus refined GK tetramer.

After obtaining active protein, we prepared cryo-samples of mixed-state AtP5CS1. A map containing approximately five helical units was obtained (Fig. 2d, Extended Data Fig. 5 and Supplementary Table 1). Similar to the AtP5CS2 filament, the AtP5CS1 filament is composed of tetramers serving as the basic helical unit. Adjacent helical units interact through assembly interfaces comprising hooks. Differing from the AtP5CS2 filament, the adjacent helical units in the AtP5CS1 filament do not assemble in a completely perpendicular manner. The helical twist is slightly smaller, approximately 80°.

**Extended Data Fig. 6:**
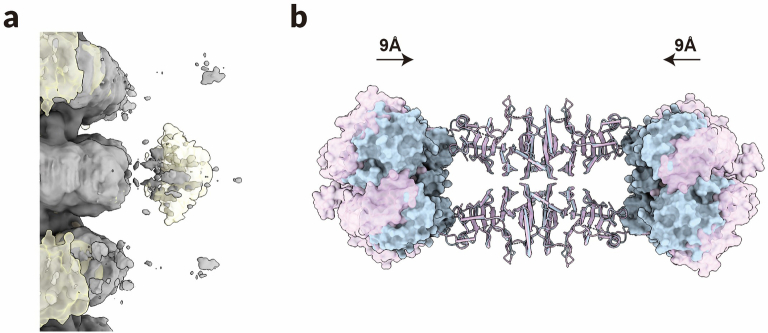
The comparison of map and model between AtP5CS1 and AtP5CS2. (**a**) Map at low contour level of AtP5CS1 and AtP5CS2. Maps of AtP5CS1 and AtP5CS2 are displayed by grey and transparent yellow respectively. (**b**) Models of AtP5CS1 and AtP5CS2 are aligned by GK tetramer and colored by blue and pink respectively. The shift of GPR along the symmetry axis is measured and indicated by the black arrow.

In the resolved density map of AtP5CS1, stable density for the GK region was obtained. However, high-resolution analysis for the GPR portion was not achieved. At lower thresholds, sparse density around the GK region was observed, potentially indicating the location of GPR (Extended Data Fig. 6a). Using AlphaFold2, we generated the full-length structure of AtP5CS1. Through fitting and manual adjustments, we built a model of the AtP5CS1 filament based on our electron density map (Fig. 2e). According to our model, the GPR domain of AtP5CS1 is positioned closer to GK. When aligning the GK tetramers, the GPR of AtP5CS1 undergoes a lateral shift of approximately 9 Å towards the centre of the tetramer in comparison to the GPR of AtP5CS2 (Extended Data Fig. 6b). From the top view, the AtP5CS1 filament presents a pattern distinct from the cross shape due to differences in the helical twist (Fig. 2f).

**Extended Data Fig. 7:**
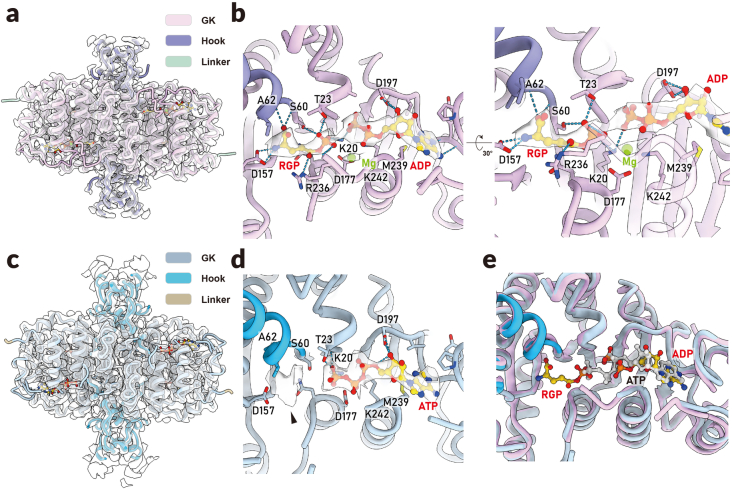
Ligands binding of AtP5CS GK domain. (**a**) Map and model of AtP5CS2 GK domain. The map generated by focus-refinement is presented by a transparent surface. The ‘hook’ region of GK domain is colored by purple. (**b**) The ligand binding pocket of AtP5CS2 GK domain. RGP and ADP, the products of GK domain are colored by gold. And their densities are displayed by the white transparent surface. Surrounding amino acids and potential interactions are indicated. (**c**) Map and model of AtP5CS1 GK domain. The map generated by focus-refinement is presented by a transparent surface. The ‘hook’ region of GK domain is colored by blue. (**d**) The ligand binding pocket of AtP5CS1 GK domain. ATP, the substrate of GK domain is colored by gold. Potential density of the substrate glutamate is also displayed, marked with black pin. (**e**) The comparison of GK pockets between AtP5CS1 and AtP5CS2.

In the GK of AtP5CS2, we captured density corresponding to adenosine diphosphate (ADP) and G5P (Extended Data Fig. 7a and Supplementary Fig. 4). Compared to ADP, RGP (PDB chemical ID for G5P) is closer to the tetramer centre, with the ammonia head of RGP stabilized by the amino acid D157, and the carbohydrate group recognized by the peptide nitrogen on the backbone from the initial part of the hook (Extended Data Fig. 7b). The gamma phosphate group of RGP has a specific interaction with the surrounding amino acids, with the side chain of amino acids S60, T23 and K20, and the backbone of the amino acid R236 involved in stabilizing the gamma phosphate group of RGP. ADP is stabilized by the ATP-binding loop of AtP5CS2. The triphosphate group of ATP is recognized and catalysed by the highly conserved functional amino acids K20, D177 and K242 of the kinase family. Besides, between the RGP and ADP, there is a magnesium ion that was previously observed in the model of DmP5CS. The conserved position for magnesium implies its potential function during the catalysis of GK.

In the model of AtP5CS1, the substrate ATP has been captured (Extended Data Fig. 7c). Similar to the ADP binding in AtP5CS2, the adenine group is stabilized by the ATP binding loop of AtP5CS1, and the triphosphate group interacts with the conserved functional amino acids K20, D177 and K242 (Extended Data Fig. 7d). We identified a small density located near the RGP binding amino acids S60, A62 and D157, implying that this could be the density of the substrate glutamate. However, due to resolution limitations, we did not model this density. When aligning the two structures, the amino acids participating in ligand recognition are conserved, and the overall conformation is similar with a root-mean-squared deviation of 0.6 Å (Extended Data Fig. 7e). The mutant variant F129A in Vigna shows the ability to eliminate the feedback inhibition of P5CS. In our model, the corresponding F128 is positioned near the centre of the AtP5CS tetramer. However, it is distanced from the catalytic pocket (Supplementary Fig. 5). The mechanism by which F128A eliminates feedback inhibition requires further experiments for a comprehensive explanation.

**Fig. 3:**
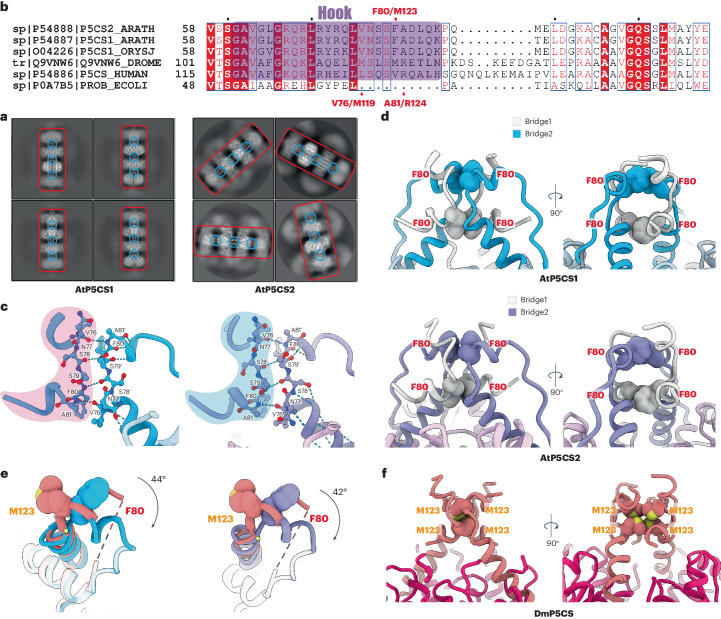
The stable interface of filament locates on the GK domain. **a**, Representative 2D classification results of AtP5CS filament. GK domain on the centre of the filament is indicated by the red box. Interfaces of GK domain composed of hooks are marked by blue circles. **b**, Sequence alignment of P5CS from different species. The hook region is indicated by a purple box. **c**, Hydrogen bond interaction inside a pair of hooks from different helical units. For clearance, one of the hooks is highlighted by a red or blue background. **d**, Front and side views of a hook tetramer in AtP5CS1 (this study) or AtP5CS2 (this study) filament. Hooks from one AtP5CS helical unit are coloured by blue or purple and the other by light grey. **e**, Comparison of hooks from AtP5CS and DmP5CS filament. By aligning helix-3 of DmP5CS and AtP5CS1 or AtP5CS2, the rotation of the hook can be measured as 44° and 42°, respectively. The rotation axis is represented as a yellow point (top view of the axis) in the figure. **f**, Front and side views of a hook tetramer in DmP5CS (PDB: 7WX3) filament.

The DmP5CS filament forms in a double helix pattern through the interaction of hooks and adjacent GPRs. However, the GPR domain of AtP5CS was found to be highly flexible based on two- and three-dimensional (2D and 3D) classification results. The representative classes of 2D classification show that the GK domain in the centre of both AtP5CS1 and AtP5CS2 remains in high resolution with clear details. A clear but small signal is resolved for the connection by the hook of adjacent helical units for both AtP5CS1 and AtP5CS2. In AtP5CS2, the GPR on the side appears in a blurred and light grey manner, while in AtP5CS1, the GPR is even more blurred and exhibits a lighter colour (Fig. 3a). These 2D classification results indicate the stable interface of AtP5CS filaments is located on the GK domain.

**Extended Data Fig. 8:**
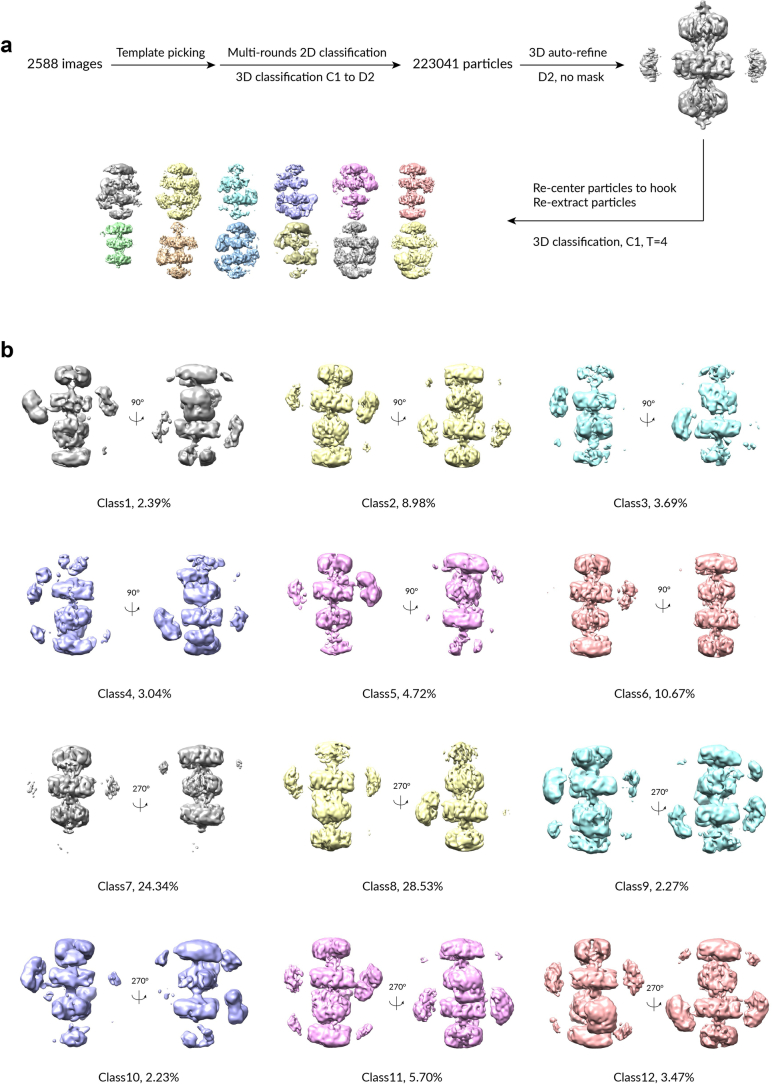
3D classification to visualize GPR of AtP5CS2. (**a**) Workflow for GPR 3D classification. After the first reconstruction for AtP5CS2, particles were re-centered to hook and re-extracted. (**b**) Display of 12 classes. GPR can be visualized in most classes with highly dynamic positions.

**Extended Data Fig. 9:**
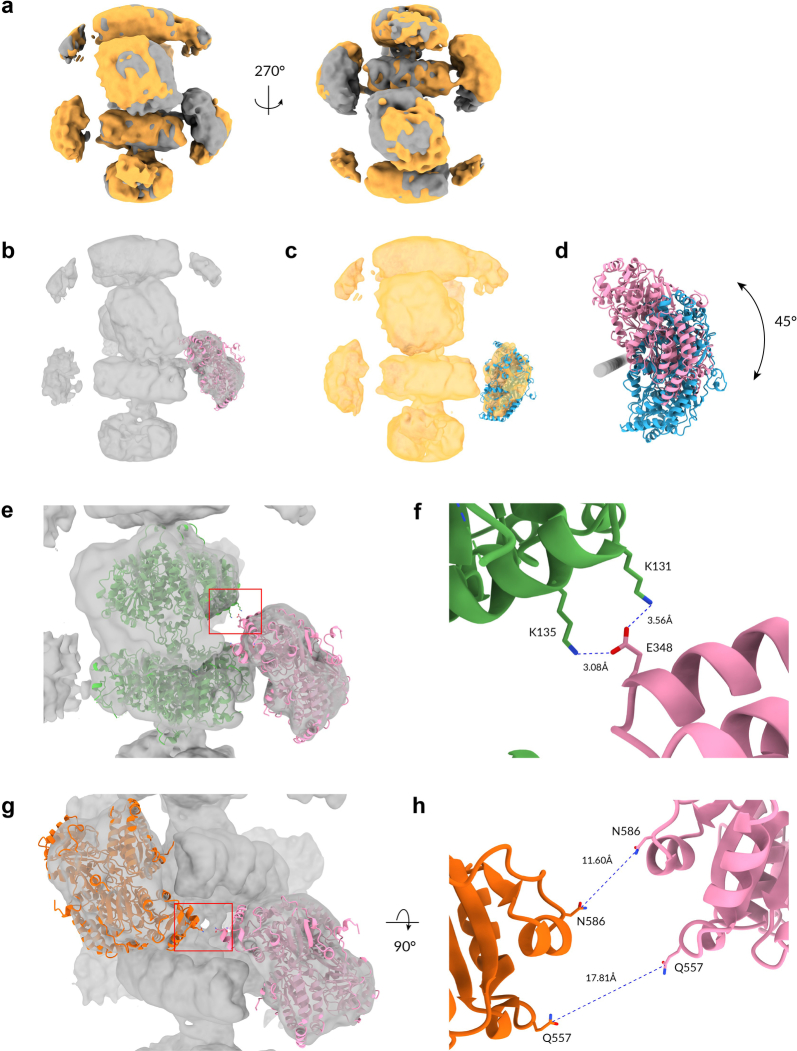
Dynamic positions of GPR dimers. (**a**) Superposition of class11 (gray) and class12 (orange) maps. GPR dimers show different locations in these two classes. (**b**) AlphaFold2 model for GPR dimer is fitted into class 11 map. (**c**) AlphaFold2 model for GPR dimer is fitted into class 12 map. (**d**) Relative rotation between GPR dimers in class 11 (pink) and class 12 (blue). The gray bar represents the rotation axis. (**e**) One GPR dimer model (AlphaFold2, pink) and two GK tetramer models (green) are fitted into class 11 map. The potential interaction region is label with red box. (**f**) Zoom in view of the red box in panel E. Rotamers of these displayed residues are manually adjusted in UCSF Chimera with Dunbrack 2010 library. (**g**) Two GPR dimer models (AlphaFold2) are fitted into class 11 map. The potential interaction region is label with red box. (**h**) Zoom in view of the red box in panel G (rotated by 90°). Rotamers of these displayed residues are manually adjusted in UCSF Chimera with Dunbrack 2010 library.

We tried to capture potential transient stable GPR locations of AtP5CS2 by 3D classification. After the particles were re-centred from the GK tetramer to the hook, the quality of 3D classification results was improved and the density of GPR dimers became solid in many classes (Extended Data Fig. 8). None of these solid GPR dimers stays at the central line of AtP5CS2 tetramer; that is, the linkers between GK and GPR are not perpendicular to the helical axis. Comparing the locations of GPR dimers in different classes, we found the GPR dimer can rotate around the GK tetramer in a considerably wide range (Extended Data Fig. 9a–d). Some transient interactions become possible during the rotation. In class 11, the most possible interaction is between GPR dimer and a GK tetramer from an adjacent helical unit. The GK tetramer model and GPR dimer model were fitted into the map. A plausible interaction between E347 and K131/135 will be seen with proper rotamers (Extended Data Fig. 9e,f). We also evaluated potential interactions between two GPR dimers. In class 11, two GPR dimers from adjacent AtP5CS2 tetramers swing toward each other (Extended Data Fig. 9g). Two pairs of Asn and Gln were found at the tip of the GPR dimer. In the fitted models, the distances between side chains of N586 pair and Q557 pair are more than 10 Å (Extended Data Fig. 9h), which is too far for direct interactions.

The sequence of the hook is conserved between AtP5CS1 and AtP5CS2 (Fig. 3b). The stable interface of both the AtP5CS1 and AtP5CS2 filaments is composed of four hooks from two adjacent helical units. Focusing on one side of the helical interface, four pairs of hydrogen bonds (V76–A81′, S78–F80′, F80–S78′ and A81-V76′) form between the backbones of a pair of hooks from adjacent helical units. This interaction provides a direct connection between adjacent helical units and is conserved in the previously determined DmP5CS structures (Fig. 3c).

In the helical interface of both AtP5CS1 and AtP5CS2, the pi–pi interaction of F80 with another F80 from the same helical unit forms a bridge-like structure. Due to the symmetry, the other two protomers from adjacent helical units also form the same structure but in an inverted orientation. Together, the two bridges combine to form a lock structure that connects and stabilizes adjacent helical units along the helical axis (Fig. 3d).

Comparing the AtP5CS and DmP5CS structures, the key residue F80 in AtP5CS has been replaced by methionine (Fig. 3b,d). There is a distinct difference in the formation of the lock structure, as the four methionines gather together and interact with each other, unlike the two bridges of a lock structure in AtP5CS that maintain distance from one another. In DmP5CS, the side chain of R124 forms an electrostatic interaction with the side chain of E116, thereby stabilizing the lock structure. However, in AtP5CS, the amino acid corresponding to R124 is replaced by the conserved alanine, and this electrostatic interaction no longer exists. This change may make the maintenance of the lock structure more reliant on the pi–pi interactions between F80s.

Multiple sequence alignment (MSA) reveals that the hook is present in both plants and animals but has many differences. Compared to animal P5CS, the following loop length of plant P5CS is noticeably shorter, and the amino acids in the relative position of F80 in plants are mainly phenylalanine, while in animals they are mainly methionine or valine (Fig. 3b). The overall conformation of the hook structure is a helix–loop–helix and exhibits a similar angle in both DmP5CS and AtP5CS. However, when aligning the long helix of the hook from AtP5CS1 and AtP5CS2 to DmP5CS, the short helix of the hook shows a rotation around the long helix of approximately 44° and 42°, respectively (Fig. 3e,f). This results in different positions of M123 and F80, and the change in the angle of the hook provides a structural basis for the difference in helical twist between DmP5CS and AtP5CSs. In DmP5CS, the contact loop of the GPR domain is necessary for filament formation. Mutation of key residues in the DmP5CS contact loop will abolish filament formation. But in AtP5CS, whether the GPR domain is necessary for filament formation is not clear. Considering the conservation in the filament interface and the potential benefits in terms of expression and purification, along with the absence of an artificial His tag in the N-terminal, we expressed and purified truncated AtP5CS2 protein consisting of residues 1–290 (referred to as AtP5CS2-GK) to explore this question.

**Fig. 4:**
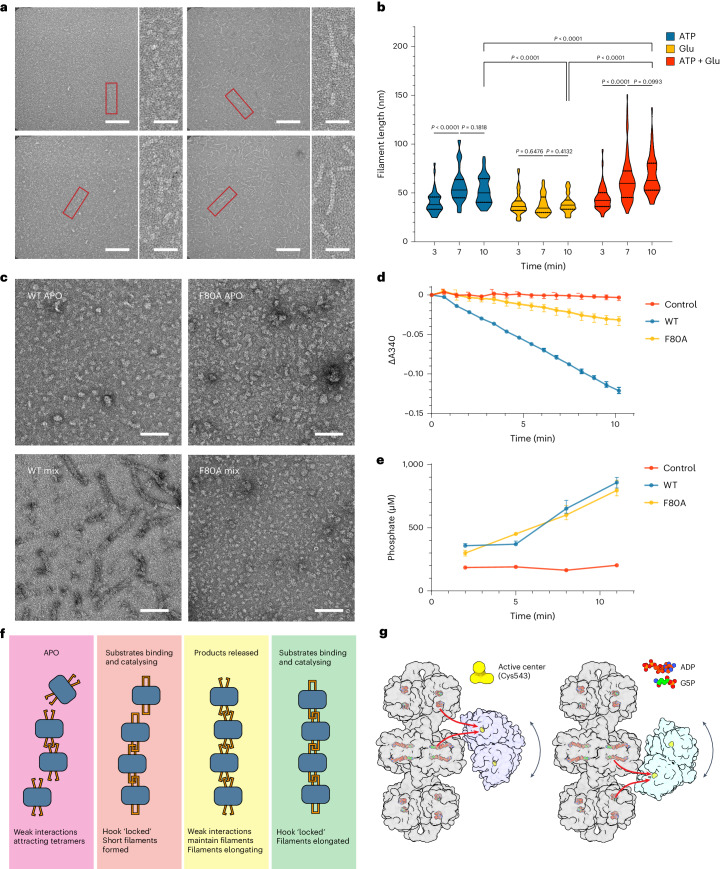
Connecting filamentation and function of AtP5CS. **a**, Negative-staining EM micrographs of AtP5CS2 GK domain incubated with different substrates. Regions in red rectangles are zoomed in on the right of the original micrographs. Scale bars, 200 nm for original micrographs and 50 nm for zoomed-in micrographs. **b**, Statistical analysis of AtP5CS2 filament length under different conditions. Violin plots show the medians (solid lines) and quartiles (dashed lines). Two-sided Mann–Whitney test was used. **c**, Negative-staining results of AtP5CS2-WT and AtP5CS2-F80A. Scale bars, 100 nm. **d**, GPR activity of AtP5CS2-WT and AtP5CS2-F80A, measured by the change of absorbance at 340 nm (A340). Error bars show the upper and lower bounds of data (*n* = 3 technical repeats). **e**, GK activity of AtP5CS2-WT and AtP5CS2-F80A, measured by phosphate generation. Error bars show the upper and lower bounds of data (*n* = 3 technical repeats). **f**, Model of AtP5CS2 filament dynamics. The blue rounded rectangles represent GK tetramers, and the orange sticks represent hooks. Two different states of hooks are shown in this figure: the open one (APO and products released states) and the locked one (substrates binding and catalysing states). **g**, Possible mechanisms of how a filament contributes to substrates channelling. Three GK tetramers (grey) and one GPR dimer (light pink or light blue) are shown here. GPR dimer can rotate up and down around the central GK tetramer, and one farthest position of GPR dimer is shown here. The activity centre of AtP5CS2 GPR is inferred from DmP5CS model (PDB: 7WXI), the key amino acid C543 for catalysing is shown. The red arrows show two possible G5P transfer paths.

Similar to full-length AtP5CS2, AtP5CS2-GK formed filaments quickly after being incubated with substrates (Fig. 4a). Few filaments were observed for APO AtP5CS2-GK (Fig. 4a). After being incubated with glutamate, only a small number of tiny filaments could be found, and 3 min incubation was enough for formation of the longest filaments. However, ATP would dramatically stimulate filament formation of AtP5CS2-GK (Fig. 4b). When ATP was present in the buffer, the average length of filaments would increase with incubation time, and the maximum length was already reached after 7 min incubation (Fig. 4b). We tested the activity of AtP5CS2-GK using Pi assay. When incubated with ATP and glutamate, Pi increased linearly with incubation time, indicating this truncated protein is enzymatically active (Supplementary Fig. 6). So, like wild-type (WT) AtP5CS2, AtP5CS2-GK forms the longest filament at reaction state.

We use site mutagenesis to study the function of AtP5CS2 filament. AtP5CS2-F80A mutant was constructed according to structural analysis. At APO state, no filament was observed for both WT and F80A AtP5CS2 (Fig. 4c). But after being incubated with all substrates, WT and mutant AtP5CS2 showed conspicuous difference (Fig. 4c). F80A mutation blocked the filament formation of AtP5CS2. We measured the overall activity of WT and F80A AtP5CS2 by decrease of absorbance at 340 nm (Fig. 4d). The decrease of absorbance at 340 nm showed good linearity within 10 min. The maximum reaction velocity (*V*_max_) of F80A mutant is 7.07 μmol of NADPH per min per μmol, only 30% of that of WT, which is 23.28 μmol of NADPH per min per μmol (Supplementary Table 2).

We tried to find how F80A mutation affected the overall activity of AtP5CS2. It has been assumed that P5CS could transfer the intermediate G5P between two domains because G5P is labile^12^. Filamentation of P5CS may facilitate the channelling of G5P. To test this hypothesis, we measured the specific activity of GK domain in WT and F80A AtP5CS2 by phosphate assay (Fig. 4e). The activity of AtP5CS2-F80A GK domain is 364.27 μmol of Pi per min per μmol, which is slightly slower than that of the WT GK domain (396.24 μmol of Pi per min per μmol). The results show that G5P generation rates are much higher than NADPH consumption rates in both WT and F80A enzymes, which is consistent with human P5CS^13^. Despite the similar specific activity of GK domain, the G5P utilization efficiency in WT enzyme (5.9%) is much higher than in the F80A enzyme (1.9%) (Supplementary Table 2). Taken together, the F80A mutation impairs the activity of AtP5CS2 by reducing the efficiency of G5P channelling.

Recently, the filament structures and regulatory mechanisms of various enzymes have been revealed^11,14–20^. In this study, we successfully expressed and purified active AtP5CS1 and AtP5CS2 proteins. By integrating the dynamic filament structures with enzyme functional assays, we propose a comprehensive model that sheds light on the intricate interplay between the catalytic and filamentation processes of AtP5CS2.

On one hand, the catalysing of AtP5CS2 serves as a driving force for filament formation. Our analyses, including negative-staining microscopy and statistical assessments, reveal that the longest filament forms during active catalysis, contrasting with the absence of filaments in the APO state. We propose a filamentation model wherein, in the APO state, specific residues surrounding the hook attract AtP5CS2 tetramers, and upon substrate binding or catalysis, these tetramers surmount the energy barrier, initializing the formation of stable filaments. The iterative interplay of attraction and catalysis processes contributes to the elongation of the AtP5CS2 filament (Fig. 4f).

**Extended Data Fig. 10:**
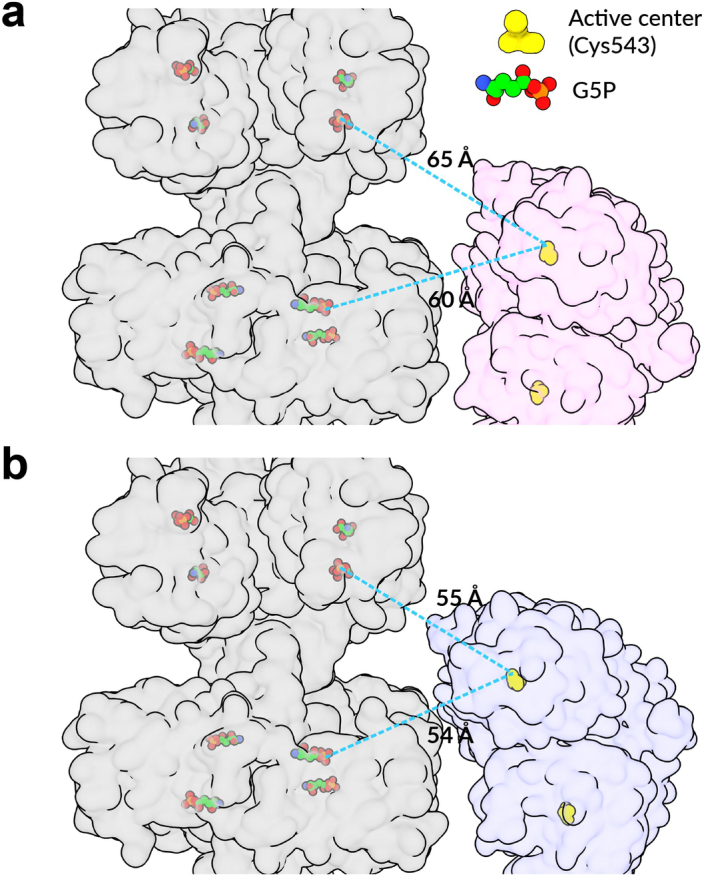
Comparison of distances between the catalytic centers of GK and GPR during the motion of AtP5CS filament. (**a**) GPR dimer locates at the ‘central position’ (AlphaFold model fitted in consensus map). The distance between catalytic centers of GK and GPR is represented by the distance of G5P and the catalytic residue C543. (**b**) GPR dimer rotates to the farthest position (AlphaFold model fitted in class 11 map). Distance measurement is the same as panel a.

On the other hand, the filamentation of AtP5CS2 plays a crucial role in enhancing the substrate channelling of G5P, facilitating the catalysis. The connection between the GK domain and GPR domain exhibits remarkable flexibility within the AtP5CS2 filament. Our 3D classification results indicate that the GPR dimer can undergo significant rotations around the GK tetramer. In certain classes of our model, compared to the ideally ‘central position’, the distance between the activity centres of GK and GPR is notably reduced (Extended Data Fig. 10). This reduction in the distance between the activity centres holds promising implications for efficient substrate channelling. Meanwhile, the distance between the GPR active site and the active site of GK from the neighbouring helical unit or the same helical unit is nearly identical, providing another potential approach for substrate transfer (Extended Data Fig. 10). The AtP5CS2 filament provides a unique platform for the rotational movement of the GPR dimer, facilitating substrate channelling (Fig. 4g).

Given the crucial role of AtP5CS1 in abiotic stress responses, the filament formation could positively impact the proline accumulation and stress responses in plants. Although AtP5CS2 filament enhances the channelling of G5P, only 5.9% G5P is used for P5C synthesis according to our enzyme activity assay (Supplementary Table 2). While the impact of AtP5CS filamentation in vivo requires further exploration, enhancing the substrate channelling efficiency of P5CS may offer a new approach for stress-resistant crop breeding.

## Methods

### Molecular cloning and site-directed mutagenesis

*A. thaliana* complementary DNA is a kind gift from B. Zheng (Fudan University). AtP5CS1 and AtP5CS2 genes were cloned into *E. coli* expression vectors using one-step cloning method (ClonExpress II One Step Cloning Kit, Vazyme #C112). The primers used for molecular cloning are provided in Supplementary Table 1. The coding sequence of AtP5CS1 gene was cloned into pET28a vector, which resulted in a His_6_-AtP5CS1 gene structure. The coding sequence of AtP5CS2 gene was cloned into a modified pET28a vector, where a SUMO-tag was fused at the N terminus of AtP5CS2, which resulted in a His_6_-SUMO-AtP5CS2 gene structure.

Site-directed mutagenesis was also performed with one-step cloning method (ClonExpress II One Step Cloning Kit, Vazyme #C112). The primers used for mutagenesis are provided in Supplementary Table 3. All plasmids generated in this study had been sequenced to guarantee correct reconstruction.

### AtP5CS1 protein expression and purification

The expression vector of AtP5CS1 was transformed into *E. coli* strain Rosetta (DE3). For protein expression, cells were first cultured at 37 °C to OD_600_ = 0.8–1.0, then protein expression was induced with 40 μM isopropylthiogalactoside at 16 °C overnight. Cells were collected by centrifugation at 4 °C and 4,000 *g*, resuspended with lysis buffer (50 mM Tris–HCl at pH 8.0, 500 mM NaCl, 10% glycerol, 20 mM imidazole, 1 mM phenylmethylsulfonyl fluoride, 5 mM β-mercaptoethanol, 5 mM benzamidine, 2 μg ml^−1^ leupeptin, and 2 μg ml^−1^ pepstatin). Then the cell suspension was homogenized with ultrasonic homogenizer (Scientz-IID) or high-pressure homogenizer (Union-Biotech LH-03). The lysate was centrifuged at 4 °C, 18,000 *g* for 45 min. Supernatant from 1 l *E. coli* medium was incubated with 1 ml Ni-NTA agarose resin (QIAGEN) for 1 h. Then the resin was washed with 10 ml wash buffer containing 40 mM imidazole (with other components the same as lysis buffer). Then proteins were eluted with 2 ml elution buffer (25 mM Tris–HCl at pH 8.0, 150 mM NaCl, 250 mM imidazole, 5 mM β-mercaptoethanol). The elution was dispensed, snap-frozen with liquid nitrogen and stored at −80 °C before use.

### AtP5CS2 protein purification

The expression vector of AtP5CS2 was transformed into *E. coli* strain Rosetta (DE3). For protein expression, cells were first cultured at 37 °C to OD_600_ = 0.8–1.0, then protein expression was induced with 40 μM isopropylthiogalactoside at 16 °C overnight. Cells were collected by centrifugation at 4 °C and 4,000 *g*, resuspended with lysis buffer (50 mM Tris–HCl at pH 8.0, 500 mM NaCl, 10% glycerol, 20 mM imidazole, 1 mM phenylmethylsulfonyl fluoride, 5 mM β-mercaptoethanol, 5 mM benzamidine, 2 μg ml^−1^ leupeptin and 2 μg ml^−1^ pepstatin). Then the cell suspension was homogenized with ultrasonic homogenizer (Scientz-IID) or high-pressure homogenizer (Union-Biotech LH-03). The lysate was centrifuged at 4 °C, 18,000 *g* for 45 min. Supernatant from 1 l *E. coli* medium was incubated with 1 ml Ni-NTA agarose resin (QIAGEN) for 1 h. Then the resin was washed with 8 ml wash buffer containing 40 mM imidazole (with other components the same as lysis buffer). Then proteins were eluted with 2–4 ml elution buffer (50 mM Tris–HCl at pH 8.0, 500 mM NaCl, 250 mM imidazole, 5 mM β-mercaptoethanol). Yeast ULP1 was used to remove SUMO-tag. About 5 ml of storage buffer (25 mM Tris–HCl at pH 8.0 and 150 mM NaCl) was added together with ULP1 for optimal ULP1 activity. After being incubated and concentrated at 4 °C, proteins were further purified through Superose 6 Increase 10/300 GL (Cytiva) in storage buffer (25 mM Tris–HCl at pH 8.0 and 150 mM NaCl). Peak fractions were collected, concentrated, dispensed, snap-frozen with liquid nitrogen and stored at −80 °C before use.

### AtP5CS activity NADPH assay

The reaction buffer contained 25 mM Tris–HCl at pH 8.0, 150 mM NaCl, 20 mM monosodium glutamate, 10 mM MgCl_2_, 5 mM ATP (Takara, sodium salt, at pH 7.0) and 0.5 mM NADPH (Roche, tetrasodium salt). Reaction was started by adding 150 nM atP5CS (wild-type or mutant). Concentration of atP5CS was determined by BCA kit (Beyotime) using BSA as reference. The reaction was monitored at 25 °C in an SpectraMax i3 plate reader (Molecular Devices). The concentration of NADPH was converted from absorbance at 340 nm with molar extinction coefficients = 6.22 l mol^−1^ cm^−1^. The path length was determined by PathCheck Sensor in SpectraMax i3 plate reader.

### AtP5CS activity Pi assay

The reaction buffer is the same as in the NADPH assay. About 150 nM enzyme was added to start the reaction. The reaction system was incubated at 25 °C. Reaction was terminated at different times by adding ice-cold 4 M perchloric acid to a final concentration of 1 M. Then perchloric acid was neutralized and precipitated with 2 M ice-cold KOH (final molar ratio perchloric acid to KOH = 1:1). The reaction mixture was centrifuged at 4 °C, 13,000 *g* for 15 min. The supernatant was used to determine Pi concentration with Malachite Green Phosphate Detection Kit (Beyotime).

### Negative staining

Proteins were incubated with different substrates at 25 °C before negative staining. All substrates used here had the same concentration as in the activity assay. Proteins were diluted to about 2–4 μM. After incubation, the protein samples were applied to plasma-cleaned carbon-coated EM grids (400 mech, EMCN). The grids were then washed twice in double-distilled H_2_O and stained with 1–2% uranyl formate. Negative-staining EM grids were imaged with a Talos L120C microscope (FEI).

### Cryo-EM grid preparation and data collection

For cryo-EM, purified atP5CS was diluted to approximately 5 μM in reaction buffer. The AtP5CS1 sample was incubated in reaction buffer for about 30 min on ice before vitrification. The AtP5CS2 sample was incubated in reaction buffer for about 60 min on ice before vitrification. The sample was applied on H_2_/O_2_ plasma-cleaned ANTcryo holy support film (Au300-R1.2/1.3) and then was immediately blotted for 3.0 s. The application and blot were repeated twice. Then the grid was plunge-frozen in liquid ethane cooled by liquid nitrogen using Vitrobot (Thermo Fisher) at 8 °C with 100% humidity. Images were collected on Titan Krios G3 (FEI) equipped with a K3 Summit direct electron detector (Gatan), operating in counting super-resolution mode at 300 kV with a total dose of 60 *e*^−^ Å^−^^2^, subdivided into 50 frames in 2.8 s exposure using SerialEM^21^. The images were recorded at a nominal magnification of ×22,500 and a calibrated pixel size of 1.06 Å, with defocus ranging from 0.8 to 2.5 μm.

### Data processing

For AtP5CS2, the image processing and reconstruction were performed with RELION 3.3 (refs. ^22,23^). We used MotionCor2 (ref. ^24^) and CTFFIND4 (ref. ^25^) via RELION GUI for pre-processing. Only micrographs with estimated resolution better than 5 Å were selected for further processing. We first used manual picking and 2D classification to generate 2D templates for atP5CS2 filament. Then AtP5CS2 particles were picked with these templates. Particles were cleaned with several rounds of 2D classifications and two rounds of 3D classifications with C1 symmetry and then D2 symmetry. Finally, 223,041 particles were used for 3D reconstruction with D2 symmetry, and a filament with three helical units was acquired. Due to the flexibility of AtP5CS2 filament, we performed an additional 3D refinement with a mask around GK tetramer to get better resolution. Then we used contrast transfer function (CTF) refinement^26^ and Bayesian polishing^27^ to further improve the resolution. For detailed analysis on heterogeneity of GK monomer, we first applied D2 symmetry expansion on refined particles (after CTF refinement and Bayesian polishing). Then we performed local 3D auto-refine on GK tetramer with C1 symmetry. Local resolution estimation was performed using Relion’s own algorithm, and locally filtered maps were used for model building. To visualize the GPR domain, we re-extracted particles with shift along the helical axis to re-centre particles on the hook. Then after one round of 3D classification, some transient locations of GPR dimers can be visualized.

For AtP5CS1, the image processing and reconstruction were performed with CryoSPARC v4.4.0 (ref. ^28^). We used patch motion correction and patch CTF estimation for pre-processing. Only micrographs with estimated resolution better than 5 Å were selected for further processing. We first used AtP5CS2 filament map to generate 2D templates. We used these templates for the first round of template picking. Particles were extracted and cleaned with several rounds of 2D classifications. The first reconstruction was made with homogeneous reconstruction using AtP5CS2 filament as reference. Then new templates were generated from the first reconstruction of AtP5CS1 and used for a second round of template picking. Particles were extracted and cleaned with multiple rounds of 2D classifications. Finally, 222,855 particles were kept for final reconstruction. We first performed homogeneous reconstruction and non-uniform reconstruction^29^. CTF refinement and reference-based motion correction were performed to improve the resolution. Then we used local refinement (to enable non-uniform reconstruction) with mask on central GK tetramer. The focus-refined map was used for model building.

### Model building

The initial model of atP5CS GK domain was acquired through AlpahFold2 (ref. ^30^) database (https://alphafold.com/). The initial model was fitted into maps using UCSF Chimera^31^. Models were iteratively refined using a combination of Coot^32^ and phenix.real_space_refine in Phenix^33,34^. For GPR dimer, model was generated with AlphaFold2 Multimer^35^ on our local server with full-length atP5CS dimer as input. To analyse possible interactions of AtP5CS2 GPR dimers, rotamers of some residues in the fitted models were adjusted in UCSF Chimera using Dunbrack 2010 (ref. ^36^) rotamer library. UCSF ChimeraX^37^ was also used for visualization purpose.

### Evolutional analysis of P5CS

All P5CS protein sequences were downloaded from UniProt database. All MSAs used MAFFT (v7.511) with method E-INS-i^38^. For phylogenetic tree reconstruction, we downloaded all sequences that were annotated with enzyme commission number ‘2.7.2.11’ (GK) or ‘2.7.2.11’ and ‘1.2.1.41’ (P5CS). Only reviewed GK sequences were used. The sequences were filtered with length to remove too short or too long sequences (with Excel) and re-mapped to UniRef50 reference sequences. After alignment, gaps were removed by trimAl (1.2rev59)^39^. A phylogenetic tree was built using IQTREE (v2.2)^40^, and a model was auto-selected^41^ (Q.pfam+I+I+R7 was chosen according to Bayesian information criterion). A phylogenetic tree was visualized using FigTree (1.4.4)^42^ and coloured with Adobe illustrator 2023. ESPript 3 (ref. ^43^) was used for MSA visualization. Sequence logos were generated using WebLogo 3 (ref. ^44^).

### Reporting summary

Further information on research design is available in the Nature Portfolio Reporting Summary linked to this article.

### Supplementary information

Supplementary Information Supplementary Figs. 1–6 and Tables 1–3.

Reporting Summary

## Figures and Tables

**Extended Data Fig. 2:**
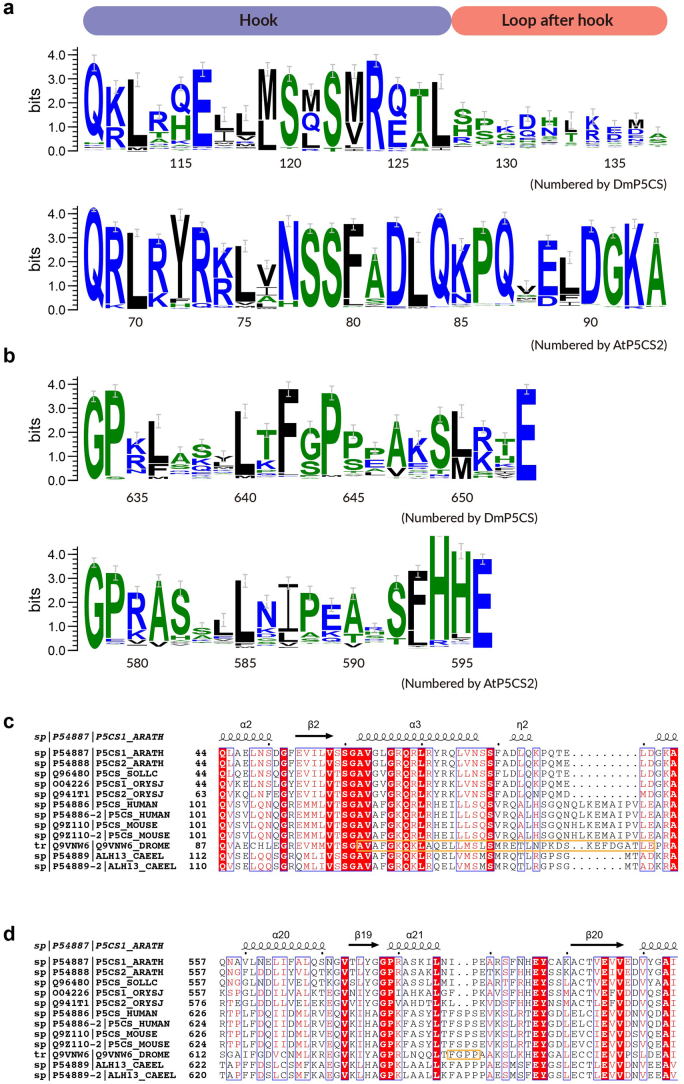
Alignment results of some representative P5CS sequences. Part of sequence logo of aligned P5CS around (**a**) hook and (**b**) contact loop. The reference residue number at the bottom is numbered based on DmP5CS and AtP5CS2. (**c**) Sequence alignment result around hook motif. Residues corresponding to hook and following loop of DmP5CS are labeled with orange box. (**d**) Sequence alignment result around contact loop. Residues corresponding to the conserved motif ‘FXPX’ in DmP5CS are labeled with orange box. Sequences used for (**c**) and (**d**) were (from top to bottom): *A. thaliana* P5CS1, *A. thaliana* P5CS2, tomato P5CS, rice P5CS1, rice P5CS2, human P5CS, human P5CS (isoform), mouse P5CS, mouse P5CS (isoform), *D. melanogaster* P5CS, *C. elegans* P5CS, *C. elegans* P5CS (isoform).

## Data Availability

The structure data accession codes are EMD-35901, EMD-38855, PDB-8J0F and PDB-8Y2H.
