# Supplementary material for: Dynamic Arabidopsis P5CS filament facilitates substrate channelling
Source: Nat Plants. 2024 May 13;10(6):880–9. doi: 10.1038/s41477-024-01697-w (PMC11208152; doi:10.1038/s41477-024-01697-w)
Supplement: Supplementary file 1 — Supplementary Figs. 1–6 and Tables 1–3. [file 41477_2024_1697_MOESM1_ESM.pdf]

nature plants

Letter

https://doi.org/10.1038/s41477-024-01697-w

Dynamic *Arabidopsis* P5CS filament facilitates substrate channelling

In the format provided by the authors and unedited

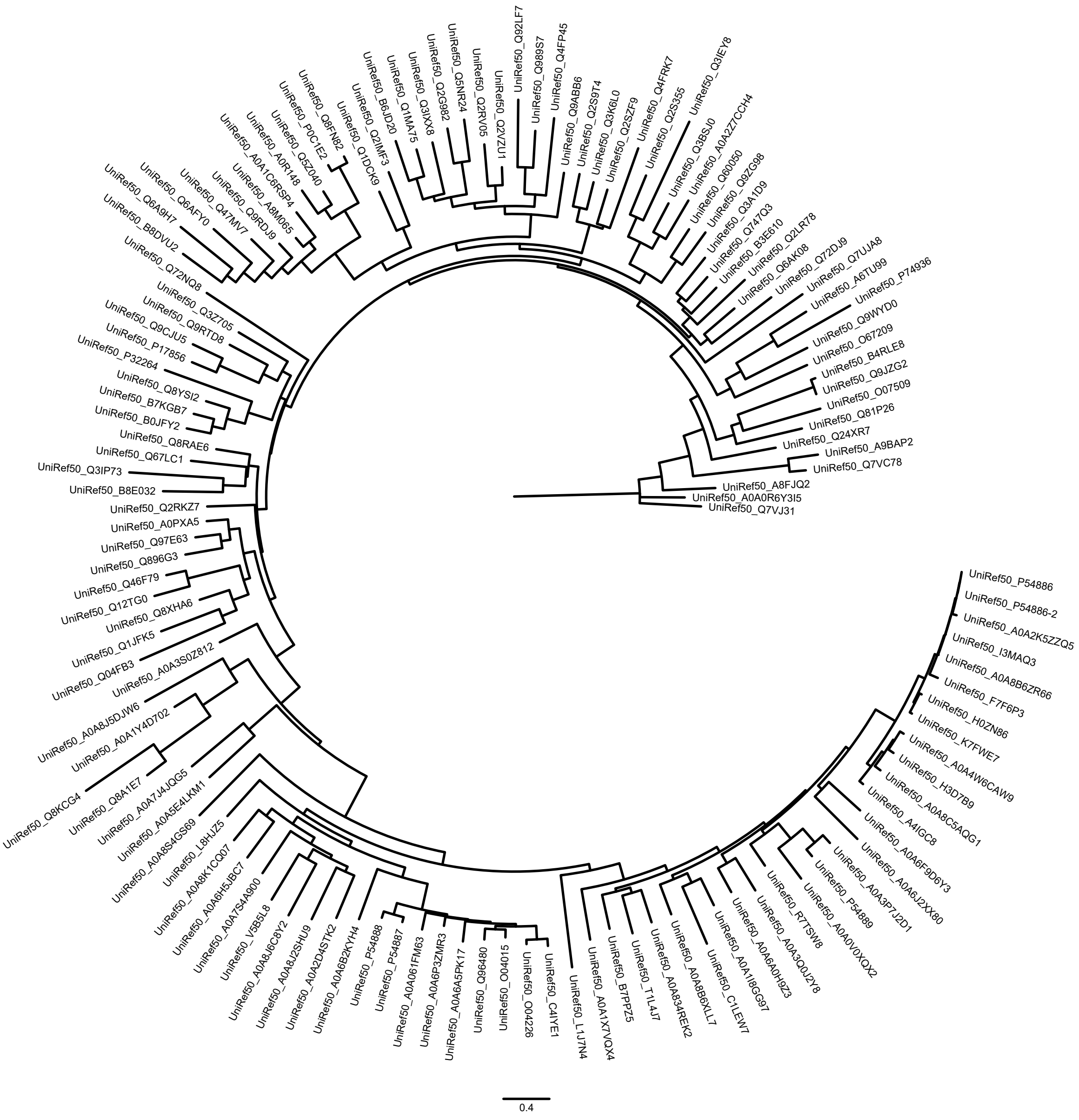

Supplementary Fig. 1: Raw phylogenetic tree of GK and P5CS.

UniRef50 representative sequences are used for multi sequence alignment and phylogenetic tree reconstruction. Name of each UniRef50 cluster is labeled at the tip of each clade. This original tree has the same orientation as the **Extended Data Fig. 1**.

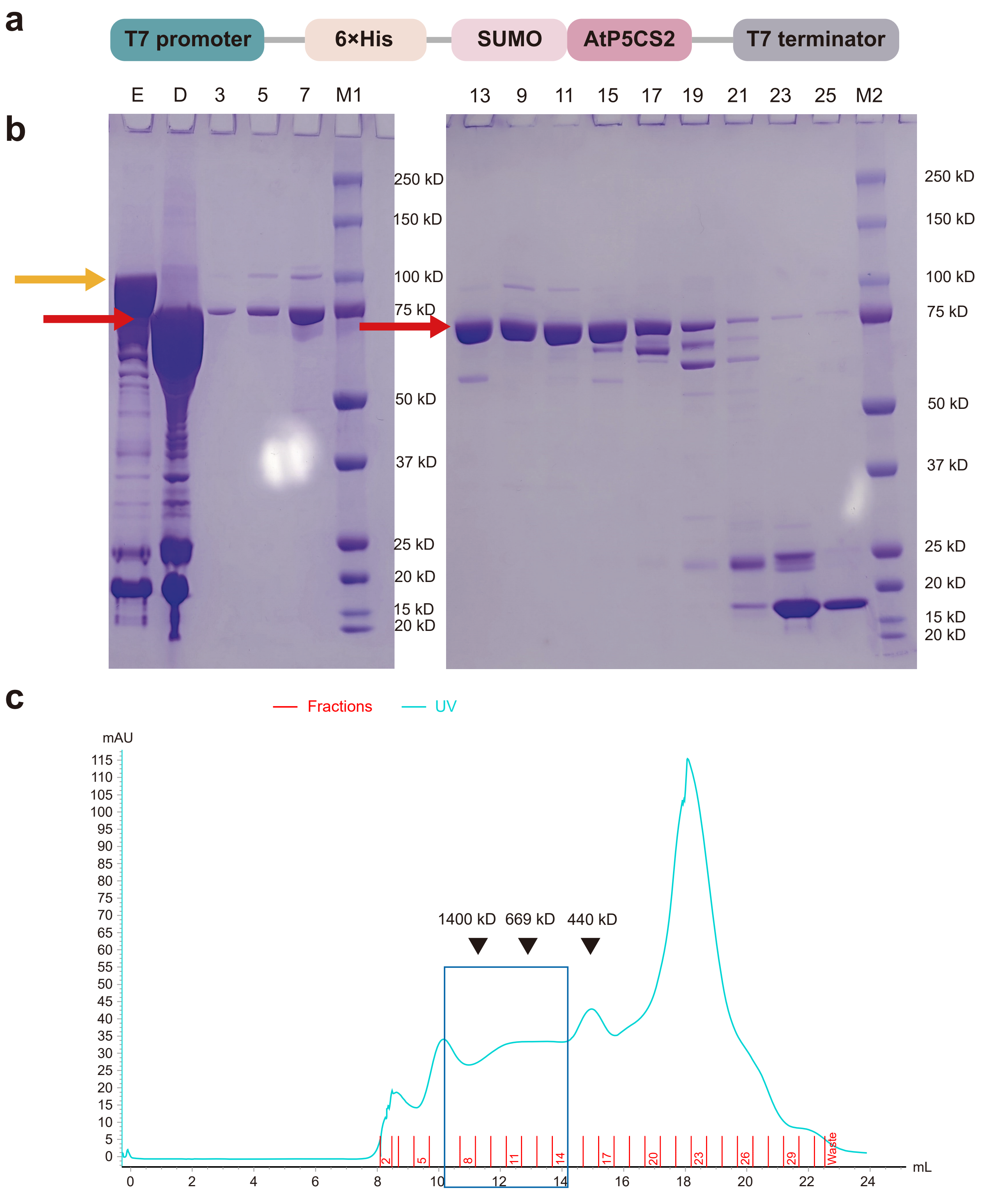

Supplementary Fig. 2: Qualification of AtP5CS2 during one representative purification experiment.

a) Gene construction for AtP5CS2 expression vector. AtP5CS2 was fused with N-terminal His_6-SUMO tag. Expression was driven by T7 promoter.

b) SDS-PAGE of AtP5CS2 at different purification stages. The band corresponding to SUMO-AtP5CS2 fusion protein (about 92.6 kDa) is indicated with yellow arrow. The bands corresponding to AtP5CS2 protein (about 78.9 kDa) are indicated with red arrows. Different fractions during purification are represented with characters on the top of panel b. "E": Elution from Ni^{2+}-NTA beads. "D": Elution after ULP1 digestion. "M1" and "M2": Standard protein markers. The corresponding molecular weights are labeled at the sides of the bands. "3" to "25": Different fractions after purification with size exclusion chromatography (Superose™ 6 Increase 10/300 GL).

c) Elution profile from size exclusion chromatography. The cyan curve indicates UV 280 nm absorbance. The fractions are numbered in red above X-axis and are the same as those in panel A. In this experiment we collected fraction 7-14 (indicated as blue box) as final product. Reference molecular weights are labeled with black triangles.

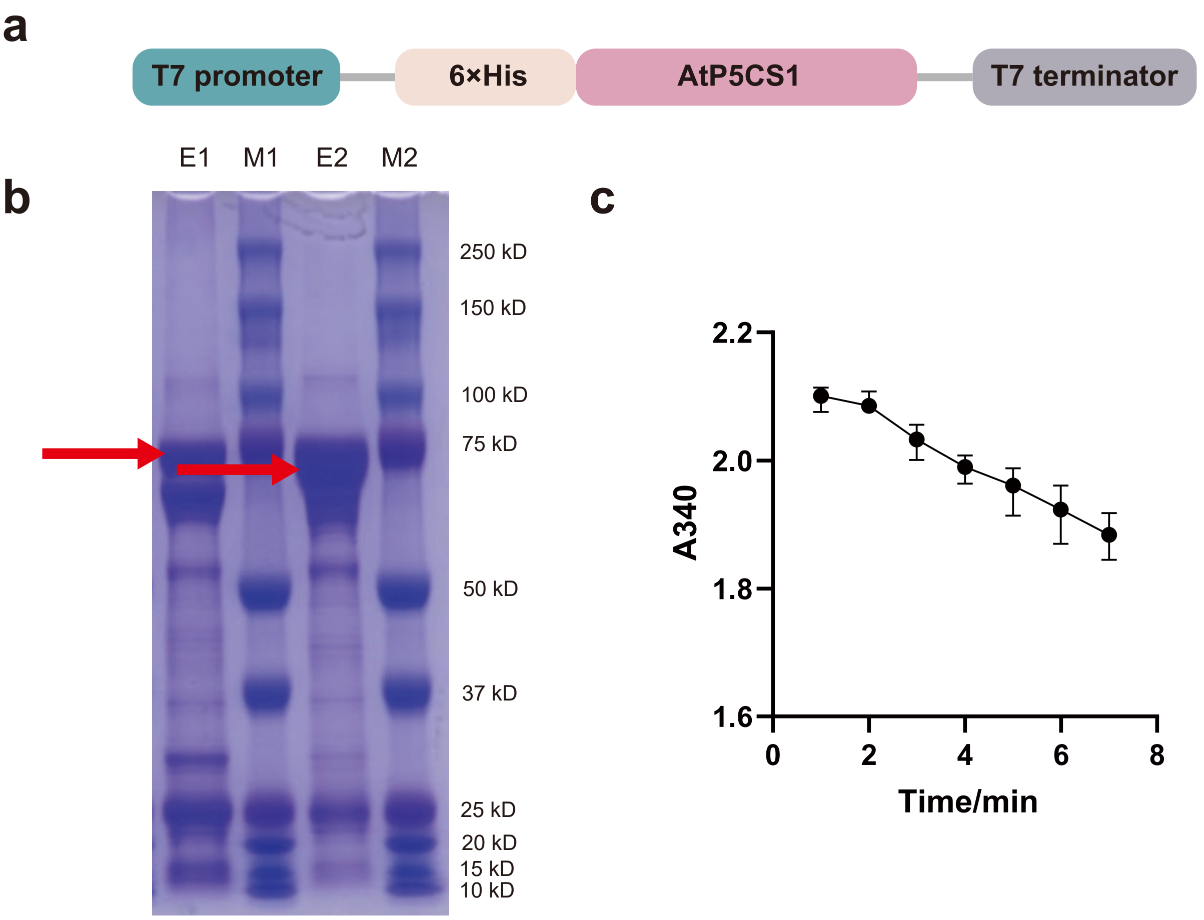

Supplementary Fig. 3: Qualification of AtP5CS1 purification.

a) Gene construction of AtP5CS1 for expression vector. AtP5CS1 was fused with N-terminal His_6-tag. Expression was driven by T7 promoter.

b) SDS-PAGE of purified AtP5CS1 products in two representative purification experiments. The band corresponding His_6-AtP5CS1 fusion protein (about 78.9 kDa) is indicated with red arrow. “E1” and “E2”: Elution from two different purification experiment. “M1” and “M2”: Standard protein markers. The corresponding molecular weights are labeled at the sides of the bands.

c) Qualification of purified AtP5CS1 by NADPH assay. The Elution “E1” was diluted by 30 times in reaction buffer. Error bar shows the upper and lower bounds of data, and n = 3 technical repeats from a same purification experiment.

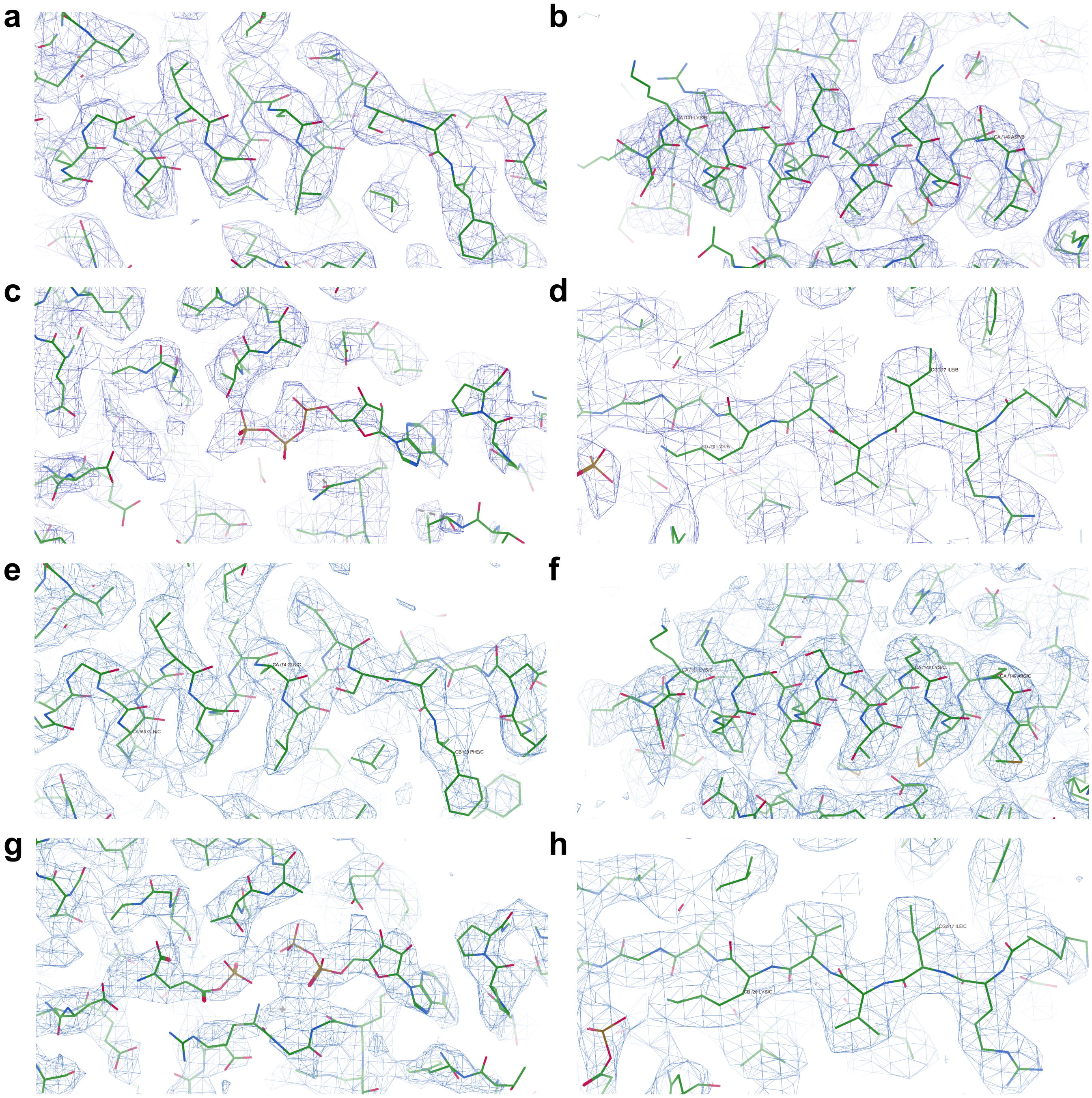

Supplementary Fig. 4: Representative density of focus refined GK tetramer.

a-d) Density of representative helixes, ligands, and β-sheet in AtP5CS1 GK tetramer map.

e-h) Density of representative helixes, ligands, and β-sheet in AtP5CS2 GK tetramer map.

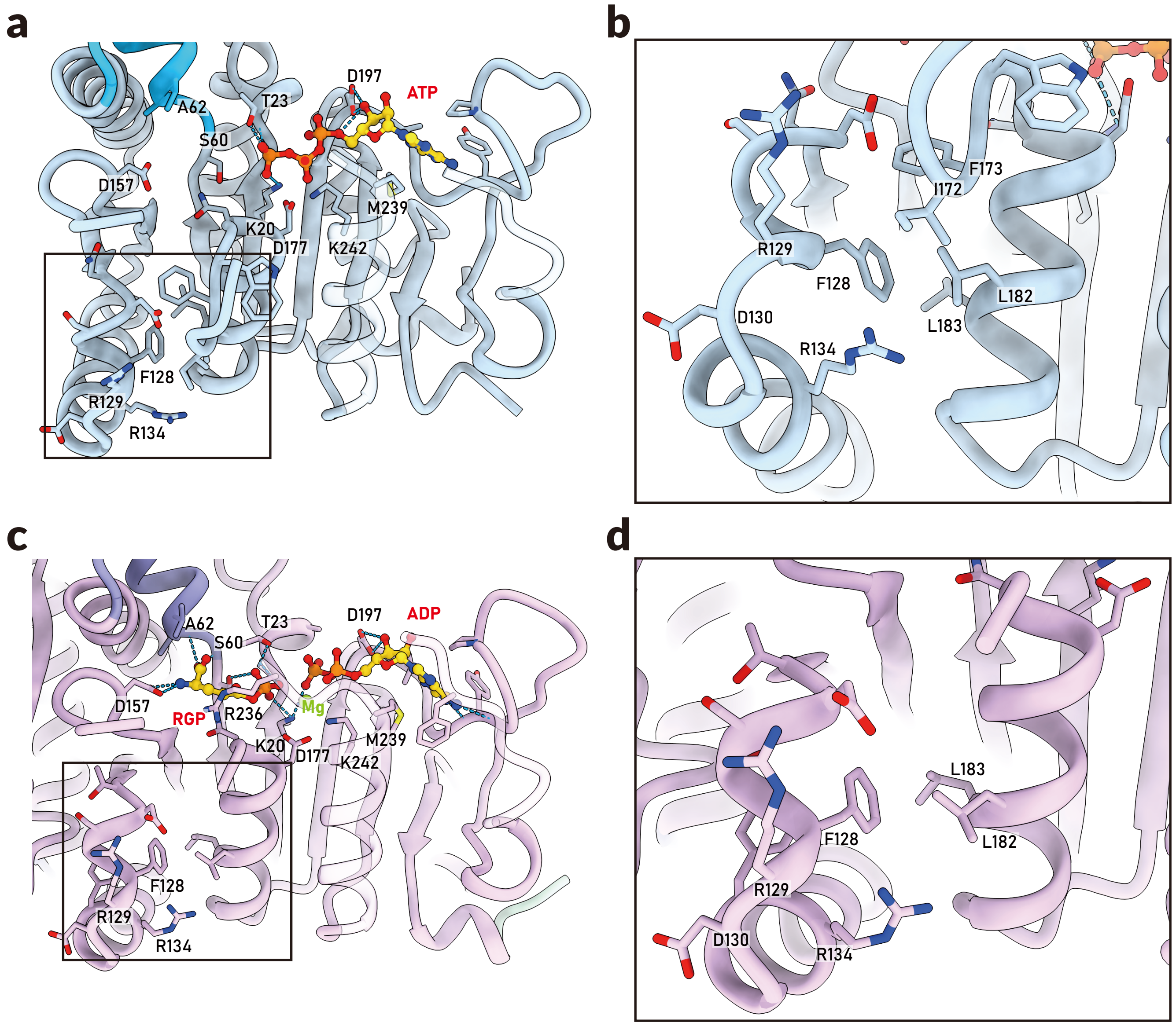

Supplementary Fig. 5: Analysis of the feedback inhibition related residue F128.

a) Location of AtP5CS1 F128, which is corresponding to VaP5CS F129.

b) Zoom-in view of F128 in panel a. F128 is buried in the hydrophobic core between helixes and is far from the catalytic center.

c) Location of AtP5CS2 F128, which is corresponding to VaP5CS F129.

d) Zoom-in view of F128 in panel c. F128 is buried in the hydrophobic core between helixes and is far from the catalytic center.

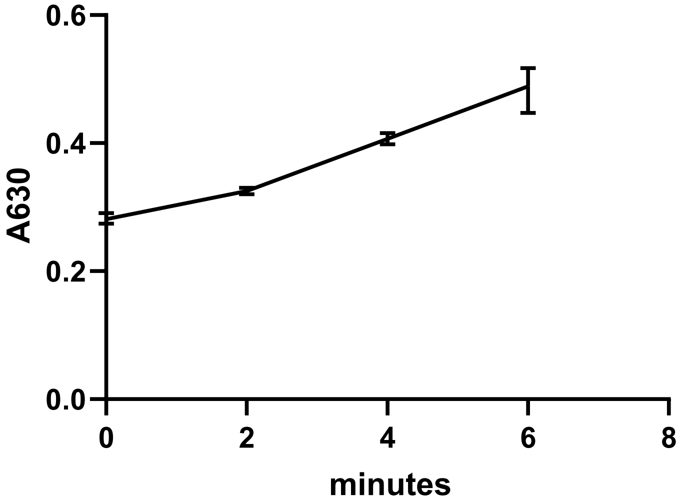

Supplementary Fig. 6: Activity of AtP5CS2-GK.

A630 is proportional to the phosphate produced in reaction buffer. Error bar shows the upper and lower bounds of data, and n = 3 technical repeats from a same purification experiment.

Supplementary Table 1 Cryo-EM data collection and model refinement

	atP5CS2 mix state (EMD-35901, PDB 8J0F)	atP5CS1 mix state (EMD-38855, PDB 8Y2H)
**Data collection**		
EM equipment	Titan Krios	Titan Krios
Detector	K3 camera	K3 camera
Magnification	22,500x	22,500x
Voltage (kV)	300	300
Electron exposure ((e–/Å^2))	60	60
Defocus range(μm)	-1.0 to -2.5	-1.0 to -2.5
Pixel size(Å)	0.53	0.53
Symmetry imposed	D2	D2
Number of collected movies	2588	4639
Initial particle images (no.)	512942	838181
Final particle images (no.)	223041	222855
Map resolution (Å)	3.3	3.3
FSC threshold	0.143	0.143
Map resolution range (Å)	3.2 - 4.3	2.8 - 6.2
**Refinement**		
Initial model used (PDB code)	7WX3	7WX3
Map sharpening B-factor(Å^2)	-120	-90
Model composition		
Non-hydrogen atoms	8956	8808
Protein residues	1208	1128
Ligands	ADP, RGP, MG	ATP
Waters	0	0
Ions	4	0
B factors(Å^2)		
Protein	148	34.3
Ligand	178	60.6
Water		
R.m.s. deviations		
Bond lengths (Å)	0.006	0.006
Bond angles (°)	0.71	0.597
Validation		
MolProbity score	1.51	1.52
Clashscore	9.13	9.9
Poor rotamers (%)	0.43	0
Ramachandran plot		
Favored (%)	97.82	98.45
Allowed (%)	2.18	1.55
Disallowed (%)	0	0

Supplementary Table 2 Domain activity of AtP5CS2-WT and atP5CS2-F80A

	WT	F80A
**GK Activity [a]**	396.24	364.27
**GPR Activity [b]**	23.28	7.07
**GPR/GK**	5.9%	1.9%

a: μmol of Pi min^{-1} μmol^{-1}. b: μmol of NADPH min^{-1} μmol^{-1}.

Supplementary Table 3 Primers used for this study.

Primer name	Sequence	Usage
pET28-AtP5CS1-F	GCCATCATCATCATCATCACATGGAGGAGCTAGATCGTTC	Clone AtP5CS1 CDS from cDNA, with homology arms.
pET28-AtP5CS1-R	TTGTTAGCAGCCGGATCTCAAGCTTGGATGGGAATGTCCTG	
pET28a-linear-F	TGAGATCCGGCTGCTAACAA	Prepare linearized pET28a vector for recombination.
pET28a-linear-R	GTGATGATGATGATGATGGC	
SUMO-AtP5CS2-F	CACAGAGAACAGATTGGTGGAATGACGGAGATCGATCGT	Clone AtP5CS2 CDS from cDNA, with homology arms.
pET28-AtP5CS2-R	GACTTAAGCATTATGCGGCCGCAAGCTAAATTCCATTCTCAACAGCCTC	
pET28a-SUMO-linear-F	TCCACCAATCTGTTCTCTGTGAGC	Prepare linearized pET28a-SUMO vector for recombination.
pET28a-SUMO-linear-R	CTTGCGGCCGCATAATGCTTAAGTC	
AtP5CS2-F80A-F	GACAATTAGTCAACAGCAGTGCTGCAGATTTACAGAAGCCAC	AtP5CS2 F80A mutagenesis.
AtP5CS2-F80A-R	GTGGCTTCTGTAAATCTGCAGCACTGCTGTTGACTAATTGTC	
