# Supplementary material for: Dynamic Arabidopsis P5CS filament facilitates substrate channelling
Source: Nat Plants. 2024 May 13;10(6):880–9. doi: 10.1038/s41477-024-01697-w (PMC11208152; doi:10.1038/s41477-024-01697-w)
Supplement: Supplementary file 2 — Reporting Summary [file 41477_2024_1697_MOESM2_ESM.pdf]

nature portfolio

Corresponding author(s): Ji-Long Liu

Last updated by author(s): Mar 14, 2024

Reporting Summary

Nature Portfolio wishes to improve the reproducibility of the work that we publish. This form provides structure for consistency and transparency in reporting. For further information on Nature Portfolio policies, see our Editorial Policies and the Editorial Policy Checklist.

Statistics

For all statistical analyses, confirm that the following items are present in the figure legend, table legend, main text, or Methods section.

n/a	Confirmed	
☐	☒	The exact sample size (*n*) for each experimental group/condition, given as a discrete number and unit of measurement
☐	☒	A statement on whether measurements were taken from distinct samples or whether the same sample was measured repeatedly
☐	☒	The statistical test(s) used AND whether they are one- or two-sided<br>*Only common tests should be described solely by name; describe more complex techniques in the Methods section.*
☒	☐	A description of all covariates tested
☒	☐	A description of any assumptions or corrections, such as tests of normality and adjustment for multiple comparisons
☐	☒	A full description of the statistical parameters including central tendency (e.g. means) or other basic estimates (e.g. regression coefficient) AND variation (e.g. standard deviation) or associated estimates of uncertainty (e.g. confidence intervals)
☐	☒	For null hypothesis testing, the test statistic (e.g. *F*, *t*, *r*) with confidence intervals, effect sizes, degrees of freedom and *P* value noted<br>*Give P values as exact values whenever suitable.*
☒	☐	For Bayesian analysis, information on the choice of priors and Markov chain Monte Carlo settings
☒	☐	For hierarchical and complex designs, identification of the appropriate level for tests and full reporting of outcomes
☒	☐	Estimates of effect sizes (e.g. Cohen's *d*, Pearson's *r*), indicating how they were calculated

Our web collection on statistics for biologists contains articles on many of the points above.

Software and code

Policy information about availability of computer code

Data collection	Unicorn (Cytiva, v7.7), SerialEM (v3), SoftMax Pro (v6.3)
Data analysis	RELION (v3.3), MotionCor2 (v1.4.5), CTFFIND4 (v4.1.10), UCSF Chimera (v1.15), UCSF ChimeraX (v1.4), AlphaFold2 (DeepMind, v2.3), Coot (v0.9.8), FigTree (v1.4.4), Phenix (v1.20.1), ImageJ (1.53), MAFFT (v7.511), MS Excel (v16.0), GraphPad Prism (v9), trimAl (1.2rev59), IQTREE (v2.2), WebLogo (v3), Espript (v3)

For manuscripts utilizing custom algorithms or software that are central to the research but not yet described in published literature, software must be made available to editors and reviewers. We strongly encourage code deposition in a community repository (e.g. GitHub). See the Nature Portfolio guidelines for submitting code & software for further information.

Data

Policy information about availability of data

All manuscripts must include a data availability statement. This statement should provide the following information, where applicable:

- Accession codes, unique identifiers, or web links for publicly available datasets
- A description of any restrictions on data availability
- For clinical datasets or third party data, please ensure that the statement adheres to our policy

The cryo-EM map generated for this manuscript is available from the EMDB (https://www.ebi.ac.uk/emdb/) with accession code EMD-35901 and EMD-38855. The

protein model generated for this manuscript is available from RCSB PDB (https://www.rcsb.org/) with accession code PDB-8J0F and PDB-8Y2H. In this study following data were used: DmP5CS filament structures (PDB: 7WX3, 7WXI).

Research involving human participants, their data, or biological material

Policy information about studies with human participants or human data. See also policy information about sex, gender (identity/presentation), and sexual orientation and race, ethnicity and racism.

Reporting on sex and gender	n/a
Reporting on race, ethnicity, or other socially relevant groupings	n/a
Population characteristics	n/a
Recruitment	n/a
Ethics oversight	n/a

Note that full information on the approval of the study protocol must also be provided in the manuscript.

Field-specific reporting

Please select the one below that is the best fit for your research. If you are not sure, read the appropriate sections before making your selection.

☒ Life sciences ☐ Behavioural & social sciences ☐ Ecological, evolutionary & environmental sciences

For a reference copy of the document with all sections, see nature.com/documents/nr-reporting-summary-flat.pdf

Life sciences study design

All studies must disclose on these points even when the disclosure is negative.

Sample size	Statistical methods were not employed to predefine sample sizes. Instead, the data size for cryo-EM was dictated by the microscope's availability and the particle density on the grids. Meanwhile, the sample sizes for functional assays and negative staining microscopy were determined by the reproducibility of recordings, typically set at 3. This n=3 parallelism adheres to the fundamental biological principle of parallelism.
Data exclusions	During cryo-EM data processing, particle images of damaged, incomplete, or contaminated structures were filtered out by both 2D and 3D classification procedures.
Replication	WT and mutant AtP5CS1 and AtP5CS2 protein were successfully purified for three times. Negative staining for each conduction was replicated for more than three times. To measure the length of P5CS filament, about 10 photographs were taken from different holes in a same EM grid, and these photographs were combined together for measurement. For enzyme activity, three successful technical replications were applied.
Randomization	For structure determination, all particles were randomly split into two groups, one for refinement and the other for validation. For the negative electron microscopy, micrographs were captured randomly.
Blinding	The investigators were not blinded to the group allocation, because no grouping was needed for this study.

Reporting for specific materials, systems and methods

We require information from authors about some types of materials, experimental systems and methods used in many studies. Here, indicate whether each material, system or method listed is relevant to your study. If you are not sure if a list item applies to your research, read the appropriate section before selecting a response.

Materials & experimental systems

n/a	Involved in the study
☒	☐ Antibodies
☒	☐ Eukaryotic cell lines
☒	☐ Palaeontology and archaeology
☒	☐ Animals and other organisms
☒	☐ Clinical data
☒	☐ Dual use research of concern
☒	☐ Plants

Methods

n/a	Involved in the study
☒	☐ ChIP-seq
☒	☐ Flow cytometry
☒	☐ MRI-based neuroimaging
